# Current and future perspectives on pregnancy and lactation-associated osteoporosis

**DOI:** 10.3389/fendo.2024.1494965

**Published:** 2024-12-05

**Authors:** Nataliya Gak, Ali Abbara, Waljit S. Dhillo, Richard Keen, Alexander N. Comninos

**Affiliations:** ^1^ Metabolic Bone Unit, Royal National Orthopaedic Hospital NHS Trust, London, United Kingdom; ^2^ Section of Endocrinology & Investigative Medicine, Imperial College London, London, United Kingdom; ^3^ Endocrine Bone Unit, Imperial College Healthcare NHS Trust, London, United Kingdom

**Keywords:** osteoporosis, pregnancy, lactation, fracture, bone density

## Abstract

Normal pregnancy and lactation have a marked physiological impact on maternal bone metabolism. This impact is usually temporary and reversible, but some women sustain fragility fractures whilst pregnant or lactating, termed pregnancy and lactation-associated osteoporosis (PLO). These fractures have severe negative consequences on their quality of life, at what is a crucial stage in a mother’s life. Identifiable risk factors include a low body mass index (BMI), reduced physical activity during adolescence, a strong family history of osteoporosis, and genetic variations in the LRP5 and WNT1 genes. However, due to the rarity of PLO and the limited awareness surrounding it, there has been slow progress in understanding its pathophysiology and identifying the most effective treatments. Indeed, the data available primarily originates from observational and case studies, resulting in little clear guidance on a comprehensive and multidisciplinary approach. This mini-review synthesises the latest data on incidence, pathophysiology, and management in PLO, providing current and future perspectives and highlights the need for evidence-based guidelines to improve both short-term and long-term outcomes for women with PLO.

## Introduction

Pregnancy and lactation-associated osteoporosis (PLO), also known as pregnancy-associated osteoporosis if purely in pregnancy (PAO), is a challenging, yet poorly understood condition which is characterised by the occurrence of fragility fractures during the last trimester of pregnancy or in the postpartum–lactation period (1, 2). Despite the severe physical and psychosocial consequences of fracturing around pregnancy, progress in the understanding and management of PLO has faced challenges due to its rarity and a consequent lack of awareness amongst clinicians and patients. To gain insight into PLO, we must first recognise the physiological changes that take place in maternal bone metabolism as well as the wider musculoskeletal system during pregnancy and lactation.

### Physiological changes in bone during pregnancy and lactation

**Figure 1:**
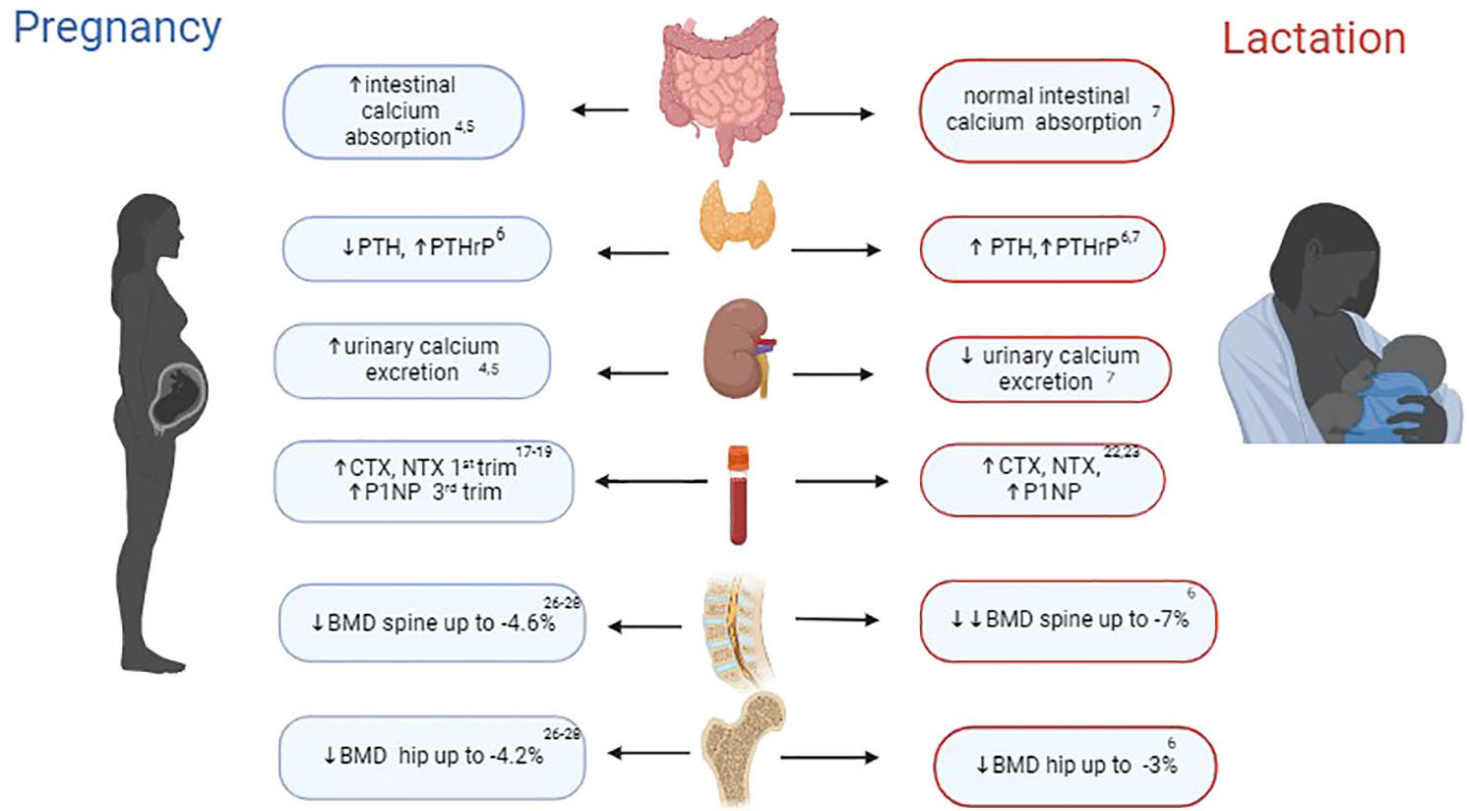
Key changes in bone physiology during pregnancy and lactation. PTH, parathyroid hormone; PTHrP, parathyroid-related protein hormone; CTX, C-terminal telopeptide of type 1 collagen; NTX, N-terminal telopeptides of type I collagen; BMD, bone mineral density. Figure was created with BioRender.com.

An average full-term foetus contains 25-30 grams of calcium with at least 80% of the calcium, phosphorus, and magnesium present in a foetal skeleton accumulated during the last trimester of pregnancy (3). To meet the foetus’ skeletal mineralisation requirements, the maternal body goes through several changes specific to pregnancy and lactation, detailed below and outlined in **Figure 1**.

*During pregnancy*, the main adaptive change that plays a crucial role in the development of the foetal skeleton is the two-fold increase in maternal gastro-intestinal calcium absorption. Placental lactogen, elevated oestradiol levels and prolactin enhance maternal renal 1α-hydroxylase activity, leading to a rise in calcitriol levels and subsequent calcium absorption (4, 5). This process is balanced by an increase in urinary calcium excretion, which is facilitated by a higher glomerular filtration rate (GFR) during pregnancy. In addition, during pregnancy, there is a decrease in the serum levels of parathyroid hormone (PTH), to the lower end of the reference range which typically returns to mid-reference range concentrations toward the end of full term (6). This reduction in PTH is due to the increase in parathyroid-related protein hormone (PTHrP), which is not measurable in the serum of non-pregnant women by routinely available intact PTH assays. Importantly, PTHrP levels remain elevated during pregnancy and lactation with secretion from the placenta, breasts, uterus, and embryonic tissues (6).

*During lactation*, physiological calcium homeostasis differs from that during pregnancy. Women who exclusively lactate lose approximately 210 mg of calcium in their expressed milk daily (7). To ensure that these mineral requirements for lactation are adequately met, a transient process of maternal bone resorption becomes the dominant adjustment in a woman’s body during this time. Hormonal changes during lactation, including elevated levels of PTHrP and prolactin, low oestradiol (due to prolactin-induced suppression of the reproductive axis) and progesterone, as well as a reduction in intestinal calcium absorption to pre-pregnancy levels (due to prompt return to non-pregnant calcitriol levels), collectively contribute to significant maternal bone mineral loss (7). The increased bone resorption is primarily driven by osteoclasts and mainly impacts trabecular bone and endocortical surfaces (8, 9). However, in parallel, bone loss during lactation can occur through a second mechanism termed osteocytic osteolysis, where osteocytes assume a similar role to osteoclasts in the remodelling of the perilacunar matrix, with concomitant expression of genes that govern osteoclast activity, including for cathepsin K (10, 11). Interestingly a very recent rodent study has identified that brain-derived cellular communication network factor 3 (CCN3) released from the hypothalamic kisspeptin neurons of lactating mothers, counteracts the aforementioned high bone resorption to ensure healthy species survival (12) and further confirms the role of kisspeptin-related signalling in bone health (13, 14).

An important aspect of lactation-associated bone loss is how quickly and fully it can be reversed once lactation is discontinued. The post lactation anabolic phase is largely driven by the decrease in circulating PTHrP along with the resumption of menstrual cycles and associated estradiol levels (as the previously raised prolactin which was suppressing the reproductive axis returns to pre-pregnancy levels). This decrease in PTHrP and increase in circulating oestradiol result in a reduction in receptor activator of nuclear factor-kappa-beta ligand (RANKL) signalling. This decrease in RANKL signaling contributes to osteoclast apoptosis and a reduction of bone resorption following weaning (15). Indeed, animal studies suggest that osteoclast inactivation and apoptosis occur within 2 days of scheduled weaning and are associated with transient rise in calcitriol, oestradiol and a simultaneous decline in PTH and prolactin (15, 16). Concomitantly, a surge in bone formation during weaning can be attributed to the dramatic increase in osteoblast numbers as result of an influx of osteoblast precursor cells in bone marrow (15).

### Changes in bone turnover markers and bone mineral density during pregnancy and lactation

The aforementioned bone and mineral changes that occur during pregnancy and lactation are reflected by changes in bone turnover markers and bone mineral density. Bone resorption markers rise through pregnancy, while markers of bone formation remain low in early pregnancy but increase to the upper end of the reference range by term (17–19). The higher level of bone resorption during the first trimester of pregnancy is demonstrated by iliac crest bone biopsies in pregnant women. Thereafter as pregnancy advances, bone formation is restored and predominates until delivery (20). This two-phase bone remodelling effect during pregnancy is also evidenced through reduced bone volume, which recovers through the formation of new trabeculae bone towards term (21).

Since lactation has a predominantly resorptive effect on bones, a significant increase in markers of bone resorption, exceeding the levels observed during the third trimester of pregnancy, is noticed when compared to non-lactating women and healthy controls. Interestingly, a comparable rise in bone formation, as measured by Procollagen type 1 N propeptide (P1NP) is also noted (22, 23). This elevation in P1NP levels can be attributed to the recruitment of osteoblast precursor cells by elevated circulating PTHrP, promoting osteoblast differentiation. However, this process may be followed by a pause in osteoblast differentiation at the transition from pre-osteoblasts to osteoblasts. This mechanism explains the swift rise in BMD following weaning, as the pre-osteoblasts are ready to complete their differentiation promptly once PTHrP levels diminish as a result of weaning. Once weaning is established, bone resorption markers decrease rapidly while raised bone formation markers gradually revert to pre-pregnancy levels within 6 months of weaning aiding bone recovery (6, 7, 22, 24).

Most imaging studies investigating the impact of pregnancy on maternal BMD have only compared BMD changes before and after pregnancy, rather than during pregnancy, due to concerns about ionising radiation (albeit minimal) during pregnancy. Therefore, there is limited data available regarding transformations of maternal BMD within pregnancy. Studies that used dual-energy X-ray absorptiometry (DXA) to assess maternal bone density show up to 4.6% and 4.2% decline of BMD in the spine and hip respectively comparing before and after pregnancy (17, 25–27).

Beyond BMD, Quantitative CT (QCT) or peripheral QCT (pQCT) and high resolution–pQCT (HR-pQCT) provide additional information on cortical and trabecular bone structure. A recent study by O Breasail and colleagues performed pQCT and HR-pQCT in pregnant women (n=46) in the first and last trimester of pregnancy and compared BMD measurements with a non-pregnant control group (n=37) (28). At the distal tibia, trabecular and cortical volumetric BMD (vBMD) was reduced by an average of 1.48% and 0.81% respectively, when measured by HR-pQCT between the first and third trimester of pregnancy. Additionally, the pregnant group experienced a significant reduction in trabecular thickness at the distal tibia (HR-pQCT), with a mean reduction of 5.3% when compared between first and third trimesters of pregnancy. At the radius endosteal resorption was only observed with pQCT at the cortical‐rich proximal site. These findings identify a differential effect of pregnancy on bone structure, depending on whether the site is weight-bearing or non-weight-bearing.

An alternative novel imaging technique is Non-ionizing Radiofrequency Echographic Multi Spectrometry (REMS). This can be safely used to monitor bone status throughout pregnancy as it does not involve ionizing radiation. Femoral neck BMD changes were assessed during pregnancy in 43 healthy pregnant women during the first and third trimester of pregnancy. A significant mean decline of 2.07% in femoral neck BMD was observed (29).

An even higher degree of BMD reduction (measured by DXA) across various anatomical sites is observed during lactation. The most substantial decrease, averaging between 5-10%, is observed in the lumbar spine (mirroring the predominant vertebral fractures in PLO). The hip, femur, and distal radius also experience BMD declines, albeit to a lesser extent, with reductions of up to 5% (6). To date, no studies have provided additional information on bone microstructure and strength in lactating women using pQCT or HR-pQCT.

As anticipated, there is a direct correlation between the duration of breastfeeding and postpartum amenorrhea, with the extent of BMD decline. A systematic review and meta-analysis assessed women’s BMD changes during lactation (30). Lactation was linked to temporary bone loss, with potent post-lactation recovery at lumbar spine and hip when comparing lumbar spine BMD measurements between the initial and final (12–18 months postpartum) assessments, an overall weighted mean difference (WMD) of 0.067 g/cm² (95% CI 0.044–0.089 g/cm²). In contrast, there was no significant difference between the initial and final femoral neck BMD measurements (p=0.3). Overall, lumbar spine BMD recovered more rapidly than femoral neck BMD, except in women who breastfed for more than 6 months. Of note, most studies included in this meta-analysis did not account for the return of ovulation and estrogen levels to pre-pregnancy levels as factors influencing BMD recovery. Follow-up assessments considering these parameters would provide further useful data to tease out the various factors contributing to post-lactation bone recovery.

## Pathophysiology of PLO

It is evident from the above, that both normal pregnancy and lactation have significant impacts on maternal bone metabolism, with lactation imposing a more substantial strain on the women’s skeleton compared to pregnancy. Despite experiencing transient and reversible physiological transformations in the skeletal system, most pregnant women do not typically experience pathological fractures during pregnancy or lactation. If such fractures do occur, PLO should be considered and is typically defined by the occurrence of fragility fractures during the last trimester of pregnancy or during lactation.

The precise incidence of PLO is uncertain. Most recently, Kasahara and colleagues approximate the occurrence of PLO in Japan to be 460 cases per million childbirths (31). In the UK (Edinburgh) the incidence of PLO has been estimated at 6.8/100.000 pregnancies (32). Of note, these recent estimates are significantly higher than previously reported estimates of 4-8 cases per million pregnancies (33), reflecting an increasing awareness of PLO. This variation in reported incidence is likely due to the limited number of specific epidemiological studies examining PLO. Mean age of women affected by PLO is 35.7 years, with a range from 19 to 47 y.o., and a predominance of Caucasian ethnicity as reported in a systematic review by Qian and colleagues (34).

Similarly, the precise pathogenesis of PLO is also currently unclear. It is proposed that pre–pregnancy low BMD, pregnancy and lactation-associated bone loss, mechanical pressure upon the maternal spine (35) and ligament laxity caused by pregnancy-related hormonal transformations play combined roles in its pathogenesis. Risk factors resulting in pre-pregnancy low BMD have been studied. In the largest case–control study of 102 patients with PLO, low BMI (<18 kg/m^2^) and reduced physical activity during childhood or adolescence increased the likelihood of subsequent PLO (33). In addition, a strong family history of osteoporosis has been identified in patients with PLO. Indeed, Peris and colleagues conducted a study exploring the presence of a family history of osteoporosis in women with PLO. The BMD of 15 relatives of five PLO patients was measured, identifying that 53% of these relatives had osteoporosis, in contrast to only 15% in a healthy control group. Notably, within the study, three specific families had multiple members with BMD in the osteoporotic range, suggesting a potentially significant genetic factor contributing to the occurrence of PLO (36). Similarly, Dunne et al. identified a higher occurrence of fragility fractures at a younger age among the mothers of 35 women with PLO when compared to a healthy control population and Laroche and colleagues reported that 20 out of 52 women with PLO had a family history of osteoporotic fractures (37, 38). These observational studies reinforce the concept that family history (suggesting genetic factors) plays an important role in PLO pathogenesis. Taking this further, a study in Germany investigated the presence of genetic factors and their potential relationship with PLO in 42 women. In 50% of the women (21 out of 42), relevant genetic mutations were identified, with the most frequently affected genes being LRP5, WNT1, and COL1A1/A2. Most cases (11 out of 21) exhibited significant genetic variations in the LRP5 and WNT1 genes. Crucially, these genes are associated with reduced bone turnover contributing to the development of early-onset osteoporosis even in non-PLO settings (39). 3 out of 21 cases had mutations in the COL1A1 and COL1A2 genes. Interestingly, genetic analysis in one patient revealed a pathogenic mutation in the ALPL gene, consistent with hypophosphatasia. The remaining 21 out of 42 cases did not exhibit any pathogenic variants in the examined genes (40).

Additionally, reproductive disorders like “absent, scanty, and rare menstruation” (10%), “female infertility due to anovulation” (30%), and “unspecified female infertility” (36%), are notably more prevalent in a PLO group (n=56) when compared to a healthy control group (n=500) (31). The observation that ovulatory disorders are linked to PLO occurrence is not unexpected as low estrogen levels result in net bone loss.

In terms of histopathological findings, bone biopsies in 7 women with PLO performed at 12 months postpartum and at least 6 months after the cessation of lactation, demonstrated a lower rate of bone formation and remodeling when compared to the bone biopsies of both idiopathic osteoporosis and healthy control women. Despite observed low bone formation in PLO patients, osteoblast numbers did not vary significantly with other non-PLO idiopathic osteoporosis and healthy controls (41), suggesting a defect in osteoblast function.

Bone microarchitecture in treatment naive PLO women (n=7) was evaluated in a retrospective study using HR-pQCT. The mean time from the fracture occurrence to evaluation was 18 months and results were compared with healthy lactating women (n=8). Women with PLO had a 25% lower mean total vBMD (p<0.02) at the distal radius than those in the control group, with trabecular compartment affected the most (-34%, p<0.01). In the cortical compartment of the distal radius, cortical thickness and density were lower by 20% and 6% respectively in PLO women, although the difference was not statistically significant due to wide variations. Similar trends to reduction were observed at the distal tibia but were non-significant possibly owing to the tibia’s weight-bearing role affording some protection (42).

Results in keeping with these findings were reported in a prospective study that evaluated bone density at the lumbar spine (L1-L2) using central QCT (cQCT). This study compared treatment-naive cohorts of women with PLO (n=19 evaluated <12 months postpartum; n=29 evaluated >12 months postpartum) and women with non-PLO idiopathic osteoporosis (n=49) to healthy controls (n=34). Women with PLO had significant reductions in mean areal BMD (aBMD) and vBMD (aBMD 0.83 g/cm ² and vBMD 0.20g/cm ², p<0.001) at the lumbar spine compared to both non-PLO idiopathic osteoporosis (aBMD 0.94 g/cm ² and vBMD 0.23g/cm ²) and those with control groups (aBMD 1.19g/cm ² and vBMD 0.27 g/cm ²) (43).

## Clinical course of PLO

The most frequent symptoms and signs experienced by women with PLO are pain in the lower back and/or reduction in height due to vertebral fractures. Vertebral fractures are the most common presentation of PLO consistent with studies above demonstrating bone loss predominantly in the spine compared to other sites (33, 44). However, other fracture sites have been reported, including the hip, sacrum, humerus, and ribs (38, 45–47).

The recurrence of new fractures during subsequent pregnancies in women with PLO is unfortunately an important issue that should be considered for future pregnancy planning. Indeed, in a study by Kyvernitakis and colleagues, that included 107 patients followed up for 6 years, 30 women experienced a subsequent pregnancy, of whom six (20%) suffered with new PLO related fractures. In addition, having more than one fracture at the time of diagnosis was also associated with an increased risk of recurrent fracture (1). Comparable findings were supported by Laroche group, where 2 out of 7 PLO women who became pregnant again, experienced a new fracture within 36 months (38). These data highlight the importance of careful pregnancy planning and potentially intermittent bone-specific treatment between pregnancies to minimise the already high risk of future fractures.

The recovery from symptoms in women with PLO occurs at a notably slow pace, emphasising the need for better management options. Indeed, the majority (59%) of patients with PLO experience pain for over three years following the onset of fractures (1). Due to chronic pain, women with PLO frequently struggle to look after their newborn and family, have limited mobility and as result suffer with anxiety and depression (48).

Spinal PLO is the most common form; however, the most disabling non-vertebral type is known as pregnancy-related transient osteoporosis of the hip (TOH).

The mean maternal age of women affected by TOH ranges between 32-35 y. o (49–51). It typically presents in the 3^rd^ trimester of pregnancy or immediately after delivery with sudden onset of severe non -traumatic hip pain radiating to the groin, a significant reduction in hip movements causing limping and inability to walk and/or hip fractures (52).

The incidence remains unknown, as about 73 case reports (50), one retrospective case series (N=34) (51) and a case-control study (N=33) (49) on pregnancy-related TOH have been documented in the literature to date. The majority of reported pregnancy-related TOH cases are unilateral (50), with 26% (n=9) of cases described as bilateral in a case series by Toussia-Cohen (51). In addition to this, hip fractures were reported in a similar proportion of TOH cases, with 6% (2 cases) reported in the case series and 12% (4 cases) in the case-control study (49, 51).

Potential causes and pathophysiology of TOH are unclear due to its rarity and resultant lack of research. However, several predisposing risks have been identified including infrequent exercising (p ≤ 0.001), increased dental problems (p=0.023) during childhood and immobilisation for at least 1 week during pregnancy (p=0.007) (49).

The development of TOH is thought to result from a combination of bone and hormonal changes associated with pregnancy and lactation, along with mechanical stress caused by the uterus compressing the common iliac vein (making TOH distinct from PLO at other skeletal sites). This compression leads to venous hypertension, increased intramedullary bone pressure, and elevated bone turnover (53). Furthermore, pressure from the foetus on the obturator pelvic nerve may also trigger bone marrow oedema (54).

MRI, considered safe during pregnancy, is the preferred diagnostic tool for the early detection of TOH, where it reveals diffuse bone marrow oedema in the affected femoral head and neck. In several case reports, both hip and lumbar spine BMD in women with TOH were found to be 20% lower than average for age in a non- pregnant woman (55, 56). Nonetheless, neither of the larger TOH studies conducted by Hadji or Tousia-Cohen has provided mean BMD measurements of women with TOH. Management of TOH involves avoiding weight-bearing on the affected hips to reduce the risk of possible imminent hip fractures, using analgesics, avoiding breastfeeding, and considering the use of anabolic or antiresorptive medications after delivery. The course of TOH is self-limiting with resolution of symptoms and bone marrow oedema within 2-6 months (52). However, recurrence has been reported in 2 out 34 women with TOH in subsequent pregnancies (51).

## Management of PLO

Back pain secondary to vertebral fractures is typically managed with suitable analgesia and offering guidance regarding weight-bearing activities and mobility. By contrast, indications and optimal type/duration of treatment for low BMD in PLO women are currently unclear and there are no national or international guidelines currently available.

Some clinicians apply an observational approach or delay specific treatment due to the anticipated physiological recovery of BMD in women following delivery or weaning. A retrospective study in 13 women with PLO demonstrated a mean increase in bone density in the lumbar spine and total hip of 6.21% and 4.12%, respectively, between 8-18 months after delivery, compared to baseline measurements during the first 6 months post-delivery. Further follow-up at 2-4 years after delivery revealed even greater BMD recovery of 9.4% in the lumbar spine and 4.2% in the total hip (57). These data are in keeping with another retrospective study. In this study after 12 months, eight PLO patients treated with calcium and vitamin D supplements only, experienced a mean improvement in the lumbar spine and total hip BMD of 6.2% and 5.8% respectively. After 24 months, six of these patients experienced a mean improvement in the lumbar spine and total hip of 12.2% and 5.8% respectively, with the remaining two patients becoming pregnant again (2).

Women with PLO are frequently recommended to avoid lactation or to lactate for the shortest possible duration to maximise early recovery of BMD and to prevent further deterioration. Additionally, it is crucial to address insufficient or deficient serum levels of calcium and vitamin D (usually with supplements) to provide adequate substrate for bone recovery. While improvements in BMD are typically seen with these approaches, antiresorptive or anabolic agents have been widely used in PLO patients to expedite the recovery process and to prevent recurrent fractures (as imminent re-fracture risk is high following the index fracture) (58).

These treatment studies are detailed below and outlined in **Table 1**.

**Table 1:** Treatment studies in patients with PLO to date.

Authors	Study design	Number of Participant(s)	Age (mean years ± SD)	Treatment (number of patients)	Treatment duration range (months)	Change in BMD (mean)
Bisphosphonates						
O’Sullivan SM et al., 2006 (59)	Retrospective case series	9	30	alendronate, pamidronate, zoledronate + Ca/Vit D	12- 61	BMD: spine + 17.0% after 1 year and +23.0% after 2 years
Li et al., 2018 (60)	Retrospective case series	12	31 ± 5	Zoledronate (6) or Alendronate (6) + Ca/Vit D	6 - 60	BMD: spine +13.0% after 1 year, +13.7% after 2 years, +22.3% after 3 years. total hip BMD: +4.9% after 1 year, +7.4% after 2 years, +7.5% after 3 years
Laroche et al., 2017 (38)	Retrospective case series	7	n/a	n/a	24-36	BMD: spine +10.2% and femoral neck BMD +2.6% after 12 months
Denosumab						
Sanchez, Zanchetta et al., 2017 (61)	Case reports	2	33, 35	Denosumab + Ca/Vit D	12	BMD: spine + 17%, HRpQCT trabecular volume +17% and +7% at radius and tibia respectively; trabecular thickness + 21% and +13% respectively after 12 months
Stumpf, Kraus et al., 2021 (62)	Case report	1	33	Denosumab + Ca/Vit D	18	BMD: spine + 32% and total hip BMD + 11% vs baseline after 18 months
Teriparatide						
Lampropoulou-Adamidou et al., 2021 (2)	Retrospective observational cohort study	27	34 ± 5	Teriparatide + Ca/Vit D (19) or Ca/Vit D (8)	6-24	BMD spine: + 20% vs +6.2% in TPTD vs control group respectively and total hip mean BMD + 10% vs+ 5.8% in TPTD vs control group after 12 months. At 24 months, TPTD-treated patients (n=7) and controls (n=6) had a mean spine BMD + 32.9% vs +12.2%
Lee et al., 2021 (58)	Retrospective observational cohort study	33	31 ± 3	Teriparatide + Ca/Vit D (33)	12	BMD: spine +17.3%, BMD total hip + 4.6% after 12 months
Hong et al., 2018 (63)	Retrospective observational cohort study	32	31 ± 2	Teriparatide + Ca/Vit D (27) or Ca/Vit D only (5)	12	BMD: spine + 16.2% in TPTD group vs +3.5% in vit D/Ca group. Total hip BMD + 5.2% in TPTD-treated group vs 2.5% in vit D/Ca

BMD, Bone Mineral Density; Ca/Vit D, Calcium and Vitamin D; TPTD, Teriparatide; SD, Standard Deviation; n/a, not available.

### Bisphosphonates

A systematic review analysisng data from publications between 1990 and 2020 characterised clinical features and treatment options for PLO patients. The review found that out of 338 reported cases of women with PLO, 58 were treated with BPs (34). Another retrospective small case-series assessing the effect of BPs (pamidronate, alendronate and zoledronate) on BMD in 9 patients with PLO showed that lumbar spine BMD improved by 17% (*n*=4) and 23% (*n*=5) at 12 and 24 months respectively (59) A further retrospective case-series assessed the efficacy of BPs (alendronate, *n*=6, and zoledronate, *n*=6) in 12 PLO patients of Asian ethnicity found that following 12 months of treatment, BMD improved by 13% and 4.9% at lumbar spine and total hip respectively. Symptomatically, after 24 months of treatment, pain and mobility improved in 12 and 6 patients respectively. The authors, however, did not provide details about the specific symptom assessment method employed, except to mention that they gathered information on the change in symptoms through self-reported data from the patients (60).

Despite these studies with BPs, the use of BPs in women considering future pregnancy is not frequently recommended as animal studies show that bisphosphonates can accumulate in bones and cross the placenta (64). Likewise, a recent systematic review examined the outcomes of 108 pregnancies exposed to bisphosphonate treatment either before or during pregnancy. Spontaneous abortion was noted at a rate of 5.5%, which is below the 10-20% range observed in the general pregnant population. Congenital malformations were reported at 3.8%, exceeding the general prevalence of 2-3%, though the variety of malformations observed did not reveal a clear connection to BP use. Additionally, hypocalcaemia was observed in 3.7% of cases, which is a very small number of cases and is not suggestive of a connection between BP exposure and neonatal hypocalcemia. Both preterm births and low birth weight were reported at 2.8% aligning with the range observed in the general population. However, due to the absence of control cases in all but two studies, no definitive association or lack of association between bisphosphonate use and adverse neonatal outcomes could be confirmed (65).

Similarly, a case–control study with a short follow-up of 2 months post-delivery, demonstrated that the rate of observed congenital malformations in women exposed before and during pregnancy to bisphosphonates (n=36) is comparable to two control groups (n=93). These control groups consisted of women with systemic disease not treated with bisphosphonates and healthy women, unexposed to bisphosphonates. In this study, women with systemic disorders receiving bisphosphonate treatment, had an increased occurrence of neonatal complications and miscarriages, but a subgroup analysis suggested that this was due to the severity of the underlying disease and the concomitant use of disease-modifying drugs such as methotrexate and mycophenolate mofetil, rather than the use of bisphosphonates (66). These data are reassuring albeit from small case-series and individual reports.

However, the *long-term* consequences of bisphosphonates exposure *in utero* on the growth and skeletal development of children are not known. Only three case reports documented normal development of babies through to 12 months (67). Consequently, further analysis is needed with larger sample sizes and longer follow-up periods as currently largest single study included only 36 women, and the longest follow-up is 12 months. Until further evidence is available, bisphosphonate use among women of reproductive age (who may consider pregnancy) is not routinely recommended. Recent British Society of Rheumatology (BSR) guidelines suggest a pragmatic approach with the cessation of BPs 3 months in advance of pregnancy but also highlights the need for further controlled studies and longer mother/child follow-up given the long biological half-life of up to 10 years (68).

### Denosumab

Denosumab has also been used as a treatment for low BMD in women with PLO. Denosumab is a human monoclonal antibody against receptor activator of nuclear factor kB-ligand (RANKL) that prevents the binding of RANKL to RANK, resulting in a decrease in osteoclastogenesis and a reduction in the bone-resorbing activity of mature osteoclasts. In one PLO case report, there was a dramatic improvement of 32.2%, 13.0%, and 11.5% in BMD at lumbar spine, femoral neck and total hip respectively after 18 months of treatment (62). Sanchez and colleagues reported 2 PLO cases where denosumab has been proven to be effective resulting in improvement in BMD as well as clinical symptoms following one year of treatment. A 14% rise in lumbar spine BMD, marked enhancement in trabecular microarchitecture with 17% and 21% increase in trabecular volume and thickness of radius respectively (as evaluated by HRpQCT), and a subjective relief from back pain were reported (61). Denosumab has a 25.4-day half-life resulting in concentrations gradually decreasing over 4-5 months, and so doesn’t accumulate directly in the skeleton in a similar way to bisphosphonates. However, although this may be an appealing aspect in women of childbearing potential, the dramatic reversal of BMD benefits and the potential for rebound-related vertebral fractures following the cessation of denosumab is a matter of concern that should be carefully considered, especially in women who have previously sustained vertebral fractures. Furthermore, without an appropriate exit strategy from denosumab, further pregnancy would not be advised given the uncertainty of the effects of denosumab and ‘post-denosumab BMD lock-in’ bisphosphonates on a developing foetus.

### Teriparatide

Teriparatide (TPTD), a recombinant human parathyroid hormone 1–34 with an anabolic effect on bones and a half-life of 1 hour (69) is frequently a preferred choice for women with PLO. To date, only retrospective studies have been performed assessing TPT in patients with PLO. A multicenter, retrospective study demonstrated that patients with PLO who were treated with TPTD in addition to calcium and vitamin D for a duration of 12 months (n=15) versus controls (treated with calcium and vitamin D only (n=8)), achieved a mean BMD increase of 20% versus 6.2% (p<0.001) at the lumbar spine and 10% versus 5.8% at the total hip (p=0.43). At 24 months, TPTD-treated patients (n=7) and controls (n=6) achieved a mean lumbar spine BMD rise of 32.9% versus 12.2% (p=0.001) (2). A similar effect of TPTD on lumbar spine BMD was also reported retrospectively by Hong and colleagues. At 12 months mean lumbar spine BMD increased by 16.2% (p<0.001) in 27 women treated with TPTD plus calcium and vitamin D but only by 3.5% (p=0.045) in 5 patients treated with calcium and vitamin D supplements alone. In addition, BMD improved substantially by 5.4% (p=0.002) and 5.2% (p<0.001) in the femoral neck and total hip respectively in TPTD-treated women. Furthermore, women of younger age had a larger increase in lumbar spine BMD, regardless of the baseline lumbar spine BMD (63). These observed age-related positive outcomes may be attributed to the enhanced responsiveness to the anabolic effect of TPTD in a younger skeleton, characterised by increased PTH receptor expression (70). Consistent with this, initiating TPTD treatment at a younger age and a lower lumbar spine Z-score at baseline independently predicted a larger percentage increase in lumbar spine BMD in another Korean retrospective study (n=33, mean age 31 years) (58). This study also demonstrated that at 12 months, BMD of the spine and total hip improved from baseline by 17.3% and 4.6% respectively (p<0.05). After 12 months of TPTD treatment, thirteen out of 33 patients were switched to antiresorptive therapy (bisphosphonate users n=9, denosumab users n=4) for a median duration of 18 months, administered within the timeframe of 12-30 months following the discontinuation of teriparatide. The remaining 20 patients did not undergo antiresorptive treatment. All patients were given calcium and vitamin D supplements. During 2-3 years follow-up, patients not receiving antiresorptive therapy experienced an annual increase of 2.7% in lumbar spine and 0.9% in total hip BMD. Interestingly, there was no significant difference in BMD increase between this group and those not receiving antiresorptive therapy after completing TPTD treatment. These results have important clinical implications as they suggest that premenopausal women with PLO who discontinue treatment with TPTD after one year may not require sequential antiresorptive therapy as typically advised for postmenopausal women to prevent bone loss.

## Discussion and future directions

Despite the progress made to our understanding of PLO through retrospective observational studies and case reports, key uncertainties persist regarding its incidence, aetiology, and the most effective methods of treatment as below.

- The true global epidemiology of PLO is unclear. To address this issue a national/international registry dedicated to PLO patients should be established which can help in systematically identifying and tracking cases. This would permit comprehensive epidemiological studies focused on PLO to gather essential data on its occurrence, contributing factors, and management outcomes.
- Awareness regarding PLO among healthcare professionals (Primary Care Physicians, Obstetricians, Midwives, Health Visitors) should be improved, to identify and refer cases early. This would minimise delays in diagnosis and starting treatment.
- While the largest cohort of patients with PLO has been reported in Germany and France with 110 and 52 patients respectively (33, 38), there is relatively little information about PLO patients in the UK with only three case-series published, encompassing 24 (44), 16 (32), and 10 (71) patients with PLO, respectively.
- There is a known disparity in BMD among different racial groups. Black women demonstrate higher BMD in the spine and the hip when compared to white women. Conversely, Asian women exhibit lower BMD in both hip and spine regions than white women (72). These differences are underpinned by genetics, lifestyle, diet, and environmental factors. Therefore, to investigate whether similar racial differences apply in PLO, there is a need for more extensive global studies.
- Due to its rarity, there is limited knowledge about the pathophysiology of PLO and so our understanding of the underlying mechanisms remains incomplete. Women are not routinely screened for low BMD using DXA before becoming pregnant. As a result, it remains unclear whether PLO patients have initially low BMD and why only certain pregnant women experience fractures. A genetic panel analysis in one study identified LRP5, WNT1, and COL1A1/A2 as the most frequently affected genes in up to 50% of PLO patients, suggesting the existence of a hereditary condition of low bone mass with further deterioration during pregnancy and lactation due to increased calcium mobilization (40). However, this analysis specifically targeted a predefined bone gene panel instead of conducting a comprehensive genome-wide examination, therefore, the possibility of variations in other genes may be a fruitful area of future study. Nevertheless, for PLO cases, it is advisable to undertake genetic analyses to rule out undetected hereditary bone variants and provide a reliable longer term prognosis for the affected women and child.
- Despite the severe physical and psychological consequences of PLO, previous reports provide limited information about the short-term and long-term impact on physical, emotional and social well–being. A retrospective study from Germany exploring quality of life of 20 women with PLO, 18 of whom received bone-specific treatment, demonstrated that parameters of pain, mental and physical health improve by only 44% at 12 months from the time of the diagnosis. Most of the patients recruited in this study received treatment with either teriparatide (n=9), bisphosphonates (n=8) or denosumab (n=1). However, all women in this study continued to experience pain, psychological distress with a negative impact on mobility and other social aspects at 2 years follow-up (48). Although their BMD was measured at baseline and at 18 months, it was not explored if improvement in BMD translate into improvements in pain, disability and quality of life which may be a useful analysis in future studies to provide further evidence for the symptomatic and quality of life benefits of treatments.
- There are currently no established national or international guidelines for the management of PLO patients. To address this, prospective randomised controlled trials are required to define best practice and evidence-based guidelines and feed into international guideline groups. Considering the encouraging initial findings using TPTD in PLO from retrospective observational studies and case reports, this would be an important starting point with the potential to influence international practice in the management of PLO.
- There is a need for longer-term safety data from larger cohorts regarding the use of anti-resorptive drugs in pregnant or non-pregnant young women, although studies looking at this will likely continue to be only observational, due to ethical considerations.
- Romosozumab is a monoclonal antibody which by inhibiting sclerostin promotes bone formation and reduces bone resorption and has been fairly recently licensed for postmenopausal osteoporosis (73). There is a single case report describing the use of romosozumab in a woman with PLO who did not respond to and poorly tolerated TPTD (74). After 12 months of treatment with romosozumab, BMD at the lumbar spine, femoral neck and total hip improved by 23.6%, 6.2% and 11.2% respectively - similar to improvements noted in women treated with TPTD. In addition, two studies are currently underway exploring the effect of romosozumab on BMD in premenopausal women. One focuses on premenopausal women with idiopathic osteoporosis, while the other involves women with anorexia nervosa (ClinicalTrials.gov ID NCT04800367, NCT04779216). Larger, prospective studies exploring the use of romosozumab in PLO are similarly warranted.

## Conclusions

Due to the rarity of PLO and insufficient awareness among clinicians, there is a limited understanding of the condition and as a result a limited dataset beyond observational and case studies. This lack of awareness extends to the absence of guidelines for investigating and implementing a holistic, multidisciplinary treatment strategy. Consequently, these deficiencies can adversely affect and delay the physical recovery of PLO patients’ post-fracture, potentially resulting in long-term consequences such as chronic pain, disability, and negative socio-psychological impacts on the patients’ lives and their families. Future work targeting the gaps in our understanding of the pathophysiology and management of PLO, as identified above, have the potential to be life-changing for women with PLO by driving the first evidence-based guideline for the condition.
